# Inflammatory Correlated Response in Two Lines of Rabbit Selected Divergently for Litter Size Environmental Variability

**DOI:** 10.3390/ani10091540

**Published:** 2020-09-01

**Authors:** Dhekra Beloumi, Agustín Blasco, Raquel Muelas, María Antonia Santacreu, María de la Luz García, María-José Argente

**Affiliations:** 1 Institute for Animal Science and Technology, Universitat Politècnica de València, P.O. Box 22012, 46022 València, Spain; dhebel@upvnet.upv.es (D.B.); ablasco@dca.upv.es (A.B.); msantacr@dca.upv.es (M.A.S.); 2 Departamento de Tecnología Agroalimentaria, Universidad Miguel Hernández de Elche, Ctra de Beniel km 3.2, 03312 Orihuela, Spain; raquel.muelas@umh.es (R.M.); mariluz.garcia@umh.es (M.d.l.L.G.)

**Keywords:** C-reactive protein, divergent selection response, rabbits, susceptibility to diseases, welfare

## Abstract

**Simple Summary:**

Animal welfare is a priority objective for the livestock industry. Litter size environmental variability has been related to environmental sensitivity. A divergent selection experiment for environmental variance of litter size variance was carried out successfully in rabbits over thirteen generations. The low line showed a lower inflammatory response and susceptibility to infectious disorders than the high line. In conclusion, the decrease of environmental sensitivity seems to increase the adaptation of the animal to the environment, and thus, its welfare.

**Abstract:**

A divergent selection experiment for environmental variance of litter size variance was carried out in rabbits over thirteen generations. The aim of this study was to evaluate the inflammatory response in the two lines of the experiment, in order to analyse the effect of selection on susceptibility to diseases after challenging to stressful situations, such as 24 h after the first delivery. A total of 78 females were used in this study, 39 from each line. The line selected for litter size heterogeneity (the high line) showed lower white blood leukocyte count (WBC; −0.87 × 10^3^/µL), lower percentage of basophils (−0.11%), higher concentration of TNF-α (+13.8 pg/mL), and greater concentration of CRP (+38.1 µg/mL) than the line selected for litter size homogeneity (the low line). The high line had also higher concentrations of bilirubin, cholesterol, gamma-glutamyl transferase (GGT) and alkaline phosphatase (ALP) compared to the low line (difference between lines were +0.08 µmol/L, +0.14 µmol/L, +0.35 U/L and +2.4 U/L, respectively). The high line showed higher inflammatory response than the low line, in accordance with a larger susceptibility to infectious disorders. In conclusion, the line selected to increase litter size environmental variability seems to have poor capacity coping with environmental stressors. Therefore, selection for litter size environmental variability can be a useful way to improve animal welfare.

## 1. Introduction

In the last few decades, animal welfare has become a priority objective for farmers and the livestock industry [1]. Animal welfare is defined as the capacity of animals to cope with their environment [2]. Susceptibility to stress and diseases are closely related to this adaptation [3], playing an important role for the immune system in this process [4]. Inflammation is the immune system’s response that is triggered in response to microbial invasion or tissue damage in order to maintain the body’s homeostasis [5]. Inflammation is a complex process which involves a high number of molecules [6]. The cytokines interleukin-6 (IL-6) and tumour necrosis factor alpha (TNF-α), together with the acute-phase protein C-reactive protein (CRP), are mainly used as inflammatory biomarkers. Levels of these biomarkers help to detect the presence of inflammation and severity of disease [7,8,9,10]. The liver has an important role in the synthesis of acute-phase proteins, such as CRP, in response to cytokines such as IL-6, TNF-α and IL-1β [11]. Moreover, liver metabolism has been related to the inflammatory process [12]. 

In prolific species such as pigs, rabbits and mice, environmental variability in body weight and in litter size has been related to immune response and resistance to diseases (see review by Lung [13]). A divergent selection experiment for environmental variance of litter size at birth was performed in rabbits. After ten generations of selection, the lines showed a remarkable divergent response (2.7 kits^2^ in the low line vs. 4.4 kits^2^ in the high one) [14]. In a previous study, Argente [15] found lower basal levels of cortisol and CRP in females selected for litter size homogeneity (the low line) than those selected for litter size heterogeneity (the high line). After, vaccination against viral haemorrhagic disease and myxomatosis, the low line showed a better immune response than the high one [15]. This would indicate better ability to cope with environmental stressors, such as infections, in the homogenous line. 

The objective of this study was to evaluate the inflammatory response in the two lines of the divergent selection experiment for litter size environmental variance, in order to analyse the effect of selection on susceptibility to diseases after stressful situations, such as 24 h after the first delivery. For this purpose, additional inflammatory and biochemical markers to the ones studied by Argente [15] were measured in the thirteenth generation of the experiment.

## 2. Materials and Methods

### 2.1. Ethics Statement

All experimental procedures were approved by the Miguel Hernández University of Elche Research Ethics Committee, according to Council Directives 98/58/EC and 2010/63/EU (reference number 2017/VSC/PEA/00212).

### 2.2. Experiment Animals

A divergent selection experiment on environmental variability of litter size was carried out over thirteen generations. Selection was based on the phenotypic variance of litter size of each doe, after correcting litter size for both year-season and parity-lactation status (first parity, and lactating or not at mating in other parities) [14].

A total of seventy-eight primiparous female rabbits from the thirteenth generation, 39 from the line selected for litter size heterogeneity (the high line) and 39 from the line selected for litter size homogeneity (the low line), were used in this experiment. All the animals were reared in the farm of the Miguel Hernández University of Elche (Spain). The rabbits were fed a standard commercial diet (17% crude protein, 16% fibre, 3.5% fat, Nutricun Elite Gra^®^, De Heus Nutrición Animal, La Coruña, Spain). Food and water were provided ad libitum. Does were housed in individual cages (37.5 cm × 33 cm × 90 cm) under a constant photoperiod of 16 h continuous light: 8 h continuous darkness, and with controlled ventilation. The experiment took place from March to July. Table 1 shows the distributions of does and their liveweight per month. The temperature ranged from 15.2 °C to 32.1 °C. Reproduction was organized in discrete generations. All does were mated at the same age, i.e., at 18 weeks of age.

### 2.3. Blood Collection

Following the blood-sampling procedure described in [16], two blood samples of 3 mL were drawn from the central artery of each doe’s ear 24 h after the first delivery at twenty-two weeks of age. Delivery is a stressful event to does which may have an influence on haematological and biochemical parameters. The first blood sample was collected into a tube with tripotassium ethylenediaminetetraacetic acid (K3-EDTA). This sample was divided into two aliquots. One aliquot was used for haematology, and the other one was centrifuged (at 4000 rpm for 15 min) in order to determine concentrations of C-reactive protein (CRP), interleukin 6 (IL-6), tumour necrosis factor alpha (TNF-α), and cortisol. The plasma samples obtained by centrifugation were stored at −80 °C until further analysis. The second blood sample was collected into a lithium heparin tube. After centrifugation, the concentrations of bilirubin, cholesterol, alkaline phosphatase (ALP), gamma glutamyl transpeptidase (GGT), albumin (ALB), bile acid (BA), and blood urea nitrogen (BUN) were assessed.

### 2.4. Haemogram

Haematological parameters such as white blood leukocyte count (WBC) and the percentage of lymphocytes, neutrophils, monocytes, basophils and eosinophils were done by the haematology analyser Abacus Junior Vet (Diatron, Austria).

### 2.5. Assessment of C-Reactive Protein, Cytokines (Interleukin 6 and Tumour Necrosis Factor Alpha) and Cortisol

C-reactive protein (CRP) concentration was quantified using a commercially available ELISA kit for rabbits (catalogue number 2210-5; Life Diagnostics Inc., West Chester, PA, USA). Interleukine 6 (IL-6) concentration was analysed using a commercially available ELISA kit for rabbits (catalogue number CSB E06903Rb; Cusabio, Houston, TX, USA). The concentration of tumour necrosis factor alpha (TNF-α) was quantified using a commercially available ELISA kit for rabbits (catalogue number EL RB0011; Elabscience, Houston, TX, USA). Cortisol plasma concentration was performed using an available ELISA kit for rabbits (catalogue number CSB E06956Rb; Cusabio, Houston, TX, USA).

### 2.6. Assessment of Biochemical Parameters

Plasma concentrations of bilirubin, cholesterol, alkaline phosphatase, gamma glutamyl transferase, albumin, bile acid, and blood urea nitrogen were evaluated using the VetScan^®^ Mammalian Liver Profile Rotor from Abaxis Company.

### 2.7. Statistical Analysis

Data were analysed using the following model:

$$y_{\mathrm{ijk}}=\mu +{\mathrm{MS}}_{i}+L_{i}+{b\;x}_{\mathrm{ijk}}+e_{\mathrm{ijk}}$$

where MS_i_ is the month of blood sampling effect with five levels, L is the line effect with two levels (high and low line), b is the regression coefficient, x_ijk_ is the covariate weight and e_ijk_ is the residual term. Residuals were assumed to be independently normally distributed with the same variance. A Bayesian analysis was used, with bounded flat priors for all unknown parameters. Marginal posterior distributions were estimated for all unknowns using Gibbs sampling. Marginal posterior distributions of the differences between lines were computed with the program Rabbit, developed by the Institute for Animal Science and Technology (Valencia, Spain). Monte Carlo Markov chains of 60,000 iterations, with a burn-in period of 10,000, and only one out of every 10 samples was saved for inferences. Convergence was tested using the Z criterion of Geweke, and Monte Carlo sampling errors were computed using time-series procedures. Bayesian statistic gives a new approach to the description of the uncertainly against classical statistics. For example, we can provide the difference between lines (D_H-L_) and the precision of our estimation, finding the shortest interval with 95% probability of containing the true value, that can be asymmetric around the estimation (this is called the highest posterior density interval at 95% probability). Notice that in the Bayesian context, there is nothing like ‘significance’ [17], but we can calculate the actual probability of the difference between the high and low line |D_H-L_| being higher than zero; this is much more informative than P-values and significance (see Blasco [17] for details). We consider that there is enough evidence for the high and the low lines being different when the probability of this difference in absolute value |D_H-L_| is more than 90%. However, this is not a significance test, but a way to help the discussion, since we have all actual probabilities of the differences between lines and the reader can consider other probability as being relevant enough to differentiate both lines.

## 3. Results

### 3.1. Immune Parameters

Table 2 shows the features of the estimated marginal posterior distributions of the differences between lines (D_H-L_) for the haematological parameters. The high line had lower white blood leukocyte count (WBC; −0.87 × 10^3^/µL, *p* = 0.95) and lower percentage of basophils than the low line (−0.11%, *p* = 0.93). We did not observe differences between lines for the percentages of lymphocytes, neutrophils, monocytes, and eosinophils

### 3.2. Cytokines, C-Reactive Protein (CRP) and Cortisol

According to Table 3, the high line showed higher concentration of TNF-α (+13.8 pg/mL, *p* = 0.90) and greater concentrations of CRP in comparison with the low line (+38.1 µg/mL, *p* = 1.00). Concentrations of Interleukin 6 (IL-6) and cortisol were similar in both lines (*p* = 0.55 and *p* = 0.60, respectively).

### 3.3. Biochemical Parameters

Features of the marginal posterior distributions of the differences between the high and the low lines for biochemical parameters are given in Table 4. The concentrations of bilirubin and GGT were higher in the high line than the low line (*p* = 0.88 and *p* = 0.89, respectively). Cholesterol and ALP levels were also higher in the high line than in the low one (+0.14 µmol/L and +2.4 U/L, *p* > 0.90). There is some evidence of differences between lines in BA (*p* = 0.84). No differences between the high and low lines were found for ALB and BUN (*p* = 0.61 and *p* = 0.54).

## 4. Discussion

### 4.1. Immune Parameters

Haematological parameters provide valuable information on the health status of the animal. In the present study, we evaluated the haematological profile in the two lines at 24 h after the first delivery. We found that there was no difference between lines, except for WBC and the percentage of basophils. The WBC values obtained were within the normal range for rabbits (2.71 − 12.23 × 10^9^/L), reported by [18]. Total leucocyte counts are involved in the immunity reaction and defence of the organism [19,20]. Susceptible rabbits to acute infections may have a decrease in WBC count [21,22]. On the other hand, basophils are essential for linking innate and adaptive immunity [23]. Therefore, a higher basal concentration of WBC and basophils in the low line would be related to better disease resistance and good immunity. Lymphocytes and neutrophils are important for the immune system. Neutrophils provide the first line defence against infection in innate immune response [24]. Lymphocytes are involved in humoral and cell-mediated immunity response [25]. In a previous study, Argente [15] found a higher basal concentration of neutrophils and lower concentration of lymphocytes in the low line. However, no differences between lines were found in this study. We would like to note that sample collection was carried out at different environmental stress conditions, i.e., at first mating in the previous study and at 24 h after the first delivery in the current study, and that could affect the values of immunological parameters.

### 4.2. Cytokines, C-Reactive Protein (CRP) and Cortisol

Cortisol concentration was used as a biochemical indicator of stress and pain [26]. In the present study, the cortisol concentrations in the high and the low lines were higher than the normal range of cortisol concentration (2.6–3.8 μg/dL) for rabbits reported by [27]. This may be due to the moment of recording. Indeed, the samples were collected 24 h after parturition. It is known that parturition is considered one of the most stressful and painful events for the dam [28]. Such a stressful event could increase the cortisol concentration up to several hours postpartum [29]. This finding implies that both lines were under similar level of stress.

It is well known that a high level of stress leads to dysregulation of the immune system, [30] increasing predisposition to disease [31]. Inflammation is a biological response of the immune system that can be triggered by variety of factors, including pathogens and damaged cells [5]. In order to evaluate the inflammatory process in both divergent lines, i.e., their susceptibility to diseases, we assessed the plasma levels of two cytokines, interleukin-6 and tumour necrosis factor alpha, and C-reactive protein. Interleukin-6 (IL-6) is a pro-inflammatory cytokine partly produced by the combined action of IL-1β and TNF-α and has an effect on inflammation and the immune response [32]. TNF-α is a cytokine with pro-inflammatory activities produced by macrophages and have an important role in the innate defence mechanism [33]. A higher susceptibility to diseases is related to a higher concentration of TFN-α [34]. Both divergent lines were exposed to the same environment. However, the high line showed higher concentration of TNF-α than the low line. A high level of TFN-α agrees with higher inflammatory response in the high line, and therefore a higher susceptibility to diseases in this line.

C-reactive protein (CRP) is an acute-phase protein and an important etiological factor in inflammation [35]. Its production is mainly hepatic, by hepatocytes as a response to stimulation with IL-6, TNF-α and IL-1β (Reviewed by Stoner et al. [11]). Thus, CRP level in blood is considered an inflammatory biomarker [36]. Female rabbits of the high line showed greater CRP concentration. A higher basal CRP concentration in this line would confirm a higher sensitivity to disease, to the presence of chronic inflammation, and to a lesser tolerance to usual microorganisms in the farm microenvironment [6,37,38]. We note that selection for litter size environmental variability increased the difference in CPR between lines from eighth generation (5.6 µg/mL [15] to thirteenth generation (38.1 µg/mL), in agreement with a correlated response to selection for litter size environmental variability on the animal’s susceptibility to diseases.

### 4.3. Biochemical Parameters

Infection and inflammation seem to have consequences on liver metabolism in order to reduce this inflammation [39]. We assessed the biochemical indicator relationship between liver health and inflammation in both lines. Delivery is a stressful event to does which could affect to biochemical parameters concentrations. However, the levels of bilirubin, cholesterol, ALP, GCT, BA, ALB, and BUN in our lines were within the wide range of values reported in rabbits by [18,27] (3.4–8.5 µmol/L, 0.3–3.0 µmol/L, 9.1–94.6 IU/L, 2.5–14.5 IU/L, 0.7–19.6 µmol/L, 25–50 g/L, and 6.14–8.38, respectively). When we compared both lines, the concentrations of bilirubin and cholesterol were higher in the high line. According to Fan et al. [40], the increase in the concentration of cholesterol in rodents is a response to the production of inflammatory cytokines (mainly TNF-α and IL-1). Bilirubin is an endogenous antioxidant which promotes lipid peroxidation prevention [41]. Inoguchiet et al. [42] reported that bilirubin plays a protective role against chronic inflammation. Together, it seems that greater basal concentrations of cholesterol and bilirubin are related to higher sensitivity to inflammation and susceptibility to diseases in the high line. Gamma glutamyl transferase (GGT) is an inflammation regulator which increases first in the case of a hepatic disorder [43]. Females from the high line showed higher GGT concentration than those from the low line; this would suggest a liver dysfunction and high susceptibility to diseases [44]. The high line has a higher alkaline phosphatase (ALP) concentration than the low line. ALP is a good indicator of liver diseases and general health [45]. An increased concentration of ALP is an indication of liver dysfunction and a disruption in the inflammatory system [46]. These results agree with the greater susceptibility to diseases of this line.

Recently a genome-wide association study was performed on our lines, identifying several genes with functionality in the immune system and stress [47]. This finding corroborates the decisive role of the immune system in the environmental variation of litter size.

## 5. Conclusions

Our study shows the high line having higher inflammatory response under stressful situations such as 24 h after delivery, and consequently this line displays a greater susceptibility to diseases and stress. Therefore, selection for litter size environmental variability can be a useful way to improve animal welfare.

## Figures and Tables

**Table 1:** Distribution of number of does (n) and liveweight per month.

	N	Liveweight (g)
**March**	17	3395
**April**	16	3480
**May**	16	3290
**June**	19	3440
**July**	10	3475

**Table 2:** Immune parameters in female rabbits from the high and the low line.

	H	L	D_H-L_	HPD_95%_	*p*
**WBC (×10^3^/µL)**	7.48	8.35	−0.87	−1.8, 0.15	0.95
**Lymphocytes (%)**	60.2	61.9	−1.7	−10.4, 6.73	0.66
**Neutrophils (%)**	33.6	31.9	1.7	−6.52, 9.94	0.66
**Monocytes (%)**	3.5	3.16	0.34	−0.5, 1.2	0.77
**Eosinophils (%)**	2.28	2.41	−0.13	−0.67, 0.45	0.69
**Basophils (%)**	0.42	0.53	−0.11	−0.26, 0.03	0.93

H: median of the line selected for litter size heterogeneity (the high line); L: median of the line selected for litter size homogeneity (the low line); D_H-L_: differences between the high line and the low line; HPD_95%_: highest posterior density region at 95%; P: probability of the difference being >0 when D_H-L_ > 0 or being <0 when D_H-L_ < 0; WBC: white blood cells.

**Table 3:** Cytokines, C-reactive protein (CRP), and cortisol concentrations in female rabbits from the high and the low line.

	H	L	D_H-L_	HPD_95%_	*p*
**IL-6 (pg/mL)**	84.8	85.2	−0.6	−8.6, 7.2	0.55
**TNF-α (pg/mL)**	50.1	36.3	13.8	−9.2, 36.6	0.90
**CRP (µg/mL)**	85.5	47.4	38.1	15.8, 60.8	1.00
**Cortisol (ng/mL)**	24.5	25.1	−0.6	−4.7, 3.5	0.60

H: median of the line selected for litter size heterogeneity (the high line); L: median of the line selected for litter size homogeneity (the low line); D_H-L_: differences between the high line and the low line; HPD_95%_: highest posterior density region at 95%; P: probability of the difference being >0 when D_H-L_ > 0 or being <0 when D_H-L_ < 0; IL-6: interleukin 6; TNF-α: tumour necrosis factor-alpha.

**Table 4:** Biochemical parameters concentrations in female rabbits from the high and the low line.

	H	L	D_H-L_	HPD_95%_	*p*
**Bilirubin (µmol/L)**	4.74	4.66	0.08	−0.05, 0.2	0.88
**Cholesterol (µmol/L)**	1.24	1.1	0.14	−0.07, 0.34	0.91
**ALP (U/L)**	21.1	18.7	2.4	−0.92, 5.62	0.93
**GGT (U/L)**	5.98	5.63	0.35	−0.20, 0.92	0.89
**BA (µmol/L)**	3.15	2.59	0.56	−0.54, 1.68	0.84
**ALB (g/L)**	13.6	13.5	0.1	−0.77, 1.07	0.61
**BUN (µmol/L)**	7.56	7.62	−0.06	−1.06, 0.97	0.54

H: median of the line selected for litter size heterogeneity (the high line); L: median of the line selected for litter size homogeneity (the low line); D_H-L_: differences between the high line and the low line; HPD_95%_: highest posterior density region at 95%; P: probability of the difference being >0 when D_H-L_ > 0 or being <0 when D_H-L_ < 0; ALP: alkaline phosphatase; GGT: gamma-glutamyl transferase; BA: bile acid; ALB: albumin; BUN: blood urea nitrogen.
